# The associations between suicides, economic conditions and social isolation: Insights from Spain

**DOI:** 10.1371/journal.pone.0288234

**Published:** 2023-07-07

**Authors:** Carla Blázquez-Fernández, David Cantarero-Prieto

**Affiliations:** 1 Department of Economics, Universidad de Cantabria, Santander, Spain; 2 Health Economics Research Group-Valdecilla Health Research Institute (IDIVAL), Santander, Spain; 3 Santander Financial Institute–SANFI, Santander, Spain; Universidade Federal do Rio Grande do Norte, BRAZIL

## Abstract

Suicide is among the main challenges that need to be addressed in developed countries. In this paper, we analyse suicides across the 17 Spanish regions over the period 2014–2019. More precisely, our objective is to re-study the determinants of suicides focusing on the latest economic expansion period. We use count panel data models and sex stratification. A range of aggregate socioeconomic regional-level factors have been identified. Our empirical results show that: (1) a socioeconomic urban-rural suicide gaps exist; (2) there are significant gender differences, for the women a Mediterranean suicide pattern appears whereas unemployment levels have a significant importance for men, (3) social isolation factors, when significant, they show an (a priori) surprisingly positive result. We provide new highlights for suicide prevention in Spain. Precisely, it is highlighted that jointly policies by gender and attending to vulnerable groups are both necessary.

## Introduction

Suicide is a significant cause of death. Therefore, it is among the main challenges that need to be addressed worldwide. There are diverse reasons that could disentangle why people attempt or commit suicide: income-related-factors, mental-health disorders, lifestyles, or issues related to the social environment where the individual lives [1,2].

Thereby, as suggested in Santana et al. [3], many authors have proved that suicide mortality is determined by contextual factors related to both the socio-economic characteristics (such as poverty, deprivation, income and socioeconomic status or employment status) and the characteristics of the built environment (including density, urban/rural typology, access to facilities and services or mobility).

Moreover, social isolation and lack of social support may encourage individuals from suicide [4]. As an approximation from this social isolation is the living alone factor. Then, despite experiencing living alone does not necessarily mean experiencing being isolated and/or feeling lonely [5], it has been demonstrated that living alone is a substantial risk factor for both social isolation and loneliness [6].

As a result, we are concerned about those factors associated with the economic and the social background. More precisely, the objective of this article is to sight new light on the relationship between suicides, economic conditions and social isolation from Spain between 2014 and 2019, through different count panel data models. That is, the analysis is focused on the latest economic expansion period. Therefore, a period associated with a decrease in suicide rates.

The hypotheses to be tested are: i) regional, material deprivation and urbanization/rural factors have a substantial, significant link, with suicides; ii) social isolation factors characteristics matter in suicides; iii) there are significant gender differences when studying suicide determinants.

Thus, although suicide mortality rates for Spain are until the date well below the average these figures are important in order to avoid increases in the coming months/years. In fact, if possible, policies should go directed not only not to increase the number of suicides but also to reduce them. In fact, according with World Development Indicators [7] rates (defined as the total number of suicides per 100.000 population) moved from 8.6% to 7.7% between 2014 and 2019, while for the OECD countries they were 12.6% and 11.9%, respectively. Therefore, despite recent studies have analysed suicides for Spain [8–15], little evidence is still found for the latest economic expansion period. Hence, to identify who and why people are prone to suicide is fundamental.

Some features distinguish our approach from its predecessors. Initially, this paper focus on the latest economic expansion period, and so, the newest available information it is used. Secondly, the study jointly studied economic conditions and social isolation factors when focusing on a Mediterranean country. In addition, this study adds a clearer view of the current reality about suicides. All in all, our results try to provide a rigorous and systematic assessment of the public policies for the prevention and control for suicides.

The structure of the paper is as follows. Next, we present the data and methodological issues. Then, the estimation results are presented. Finally, we discuss the main outcomes and conclude.

## Material and methods

In order to analyse the different determinants through which deaths from suicide and self-harm in Spain may be affected, the latest economic expansion period (2014–2019) is studied.

Thereby, for our objective, following previous contributions [3,8], we consider a range of regional-level characteristics: (i) self-regional area characteristics which are fixed throughout the period under consideration (if the region is “foral” and so it has the greatest regulatory autonomy possible in indirect as well as direct taxation in Spain or if the region is located on the coast as a proxy for climatic characteristics); (ii) material deprivation factors (caught by an unemployment rate and by a ratio regarding the percentage of population that is at risk of poverty); (iii) urbanization/rural indicator (proxied by population density). Besides, we incorporate (iv) social isolation (here measured by living alone). Table 1 defines the variables.

**Table 1:** Variables and definitions.

Variable	Definition	Source
*suicides_total*	Total number of deaths from suicide and self-harm, total population.	Spanish National Institute of Statistics (INE).
*suicides_m*	Total number of deaths from suicide and self-harm, males.	Spanish National Institute of Statistics (INE).
*suicides_w*	Total number of deaths from suicide and self-harm, females.	Spanish National Institute of Statistics (INE).
*foral*	1 if the region is “foral” (and so has the greatest regulatory autonomy in indirect as well as direct taxes in Spain): Basque Country or Navarre Community.	Authors’ elaboration.
*north*	1 if the region is sited on the north of Spain: Asturias, Cantabria, Galicia and Basque Country.	Authors’ elaboration.
*mediterranean*	1 if the region is sited on the Mediterranean area of Spain: Andalusia, Balearic Islands, Canary Islands, Catalonia, Valencian Community and Murcia.	Authors’ elaboration.
*unemployment*	Unemployment rates.	Eurostat.
*at-risk-of-poverty*	At-risk-of-poverty rate (percentage of total population).	Eurostat.
*density*	Population density.	Eurostat.
isolation	Living alone rates.	Spanish National Institute of Statistics (INE).

*Source*: Authors’ elaboration.

As for the empirical strategy, because our dependent variable (*number of deaths from suicide and self-harm*) takes non-negative integer values and the dataset is longitudinal, the suitable framework is based on panel count data modelling. Precisely, Poisson and Negative binomial static panel models have been considered [16,17]. Then, the general specification to be analysed would be the following one:

$${Suicides}_{it}={\propto_{i}+x}_{it}^{\prime }\beta +u_{it}$$ (1)

where *x*_*it*_ is a vector of characteristics for region *i* at the *t*th observation, β is a vector of parameters to be estimated and *u*_*it*_ is the error term. Individual effects model allows for time series persistence via unobserved heterogeneity (∝_*i*_). Accordingly, the Poisson or negative binomial model would be as follows:

$$\mu_{it}\equiv E({Suicides}_{it}|x_{it},\propto_{i})=\propto_{i}\;\exp(x_{it}\beta );\;i=1,\ldots ,n,t=1,\ldots ,T$$ (2)

Then, as we are considering possible divergences by gender the above-mentioned (*Suicides*_*it*_) are used for each category (total, men, and women): *suicides_total*, *suicides_m*, *suicides_w*. In the subsequent Section, these panel models are applied.

## Results

As a first approximation to our econometric estimations, Table 2 summarises the main descriptive statistics of the analytical sample used in our estimates.

**Table 2:** Summary statistics.

Variable	Mean	S.D.	Min.	Max.
*suicides_total*	212.62	178.75	21	784
*suicides_m*	158.38	134.25	16	621
*suicides_w*	54.24	45.85	4	186
*foral*	0.12	0.32	0	1
*north*	0.24	0.43	0	1
*mediterranean*	0.35	0.48	0	1
*unemployment*	17.62	6.03	8.2	34.8
*at-risk-of-poverty*	20.36	8.20	7.7	38.8
*density*	167.55	186.74	25.6	839.7
isolation	25.92	2.31	20.28	30.54

*Notes*: 102 observations.

*Source*: Authors’ calculation.

**Fig 1:**
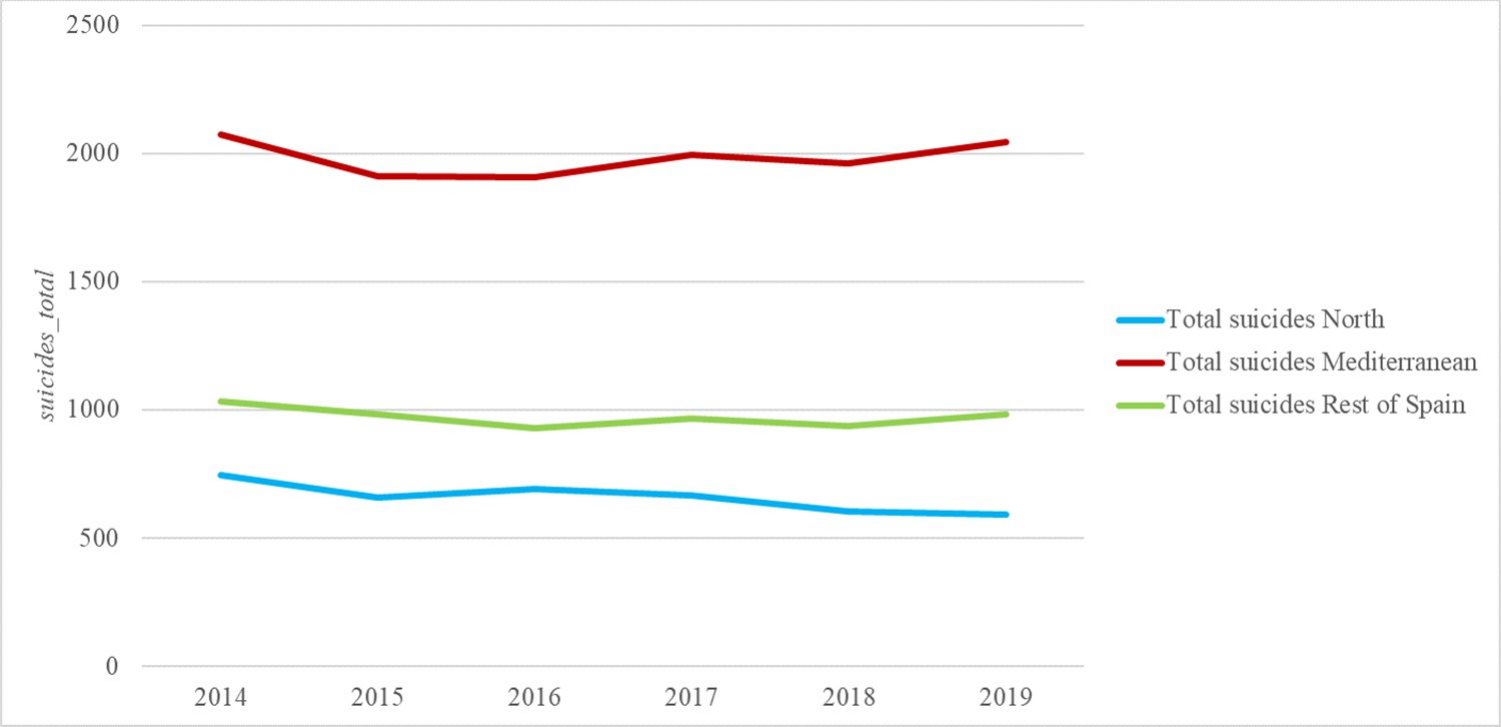
Evolution of *suicides_total* by region. *Notes*: North (Asturias, Cantabria, Galicia and Basque Country), Mediterranean (Andalusia, Balearic Islands, Canary Islands, Catalonia, Valencian Community and Murcia). *Source*: Authors’ elaboration.

**Fig 2:**
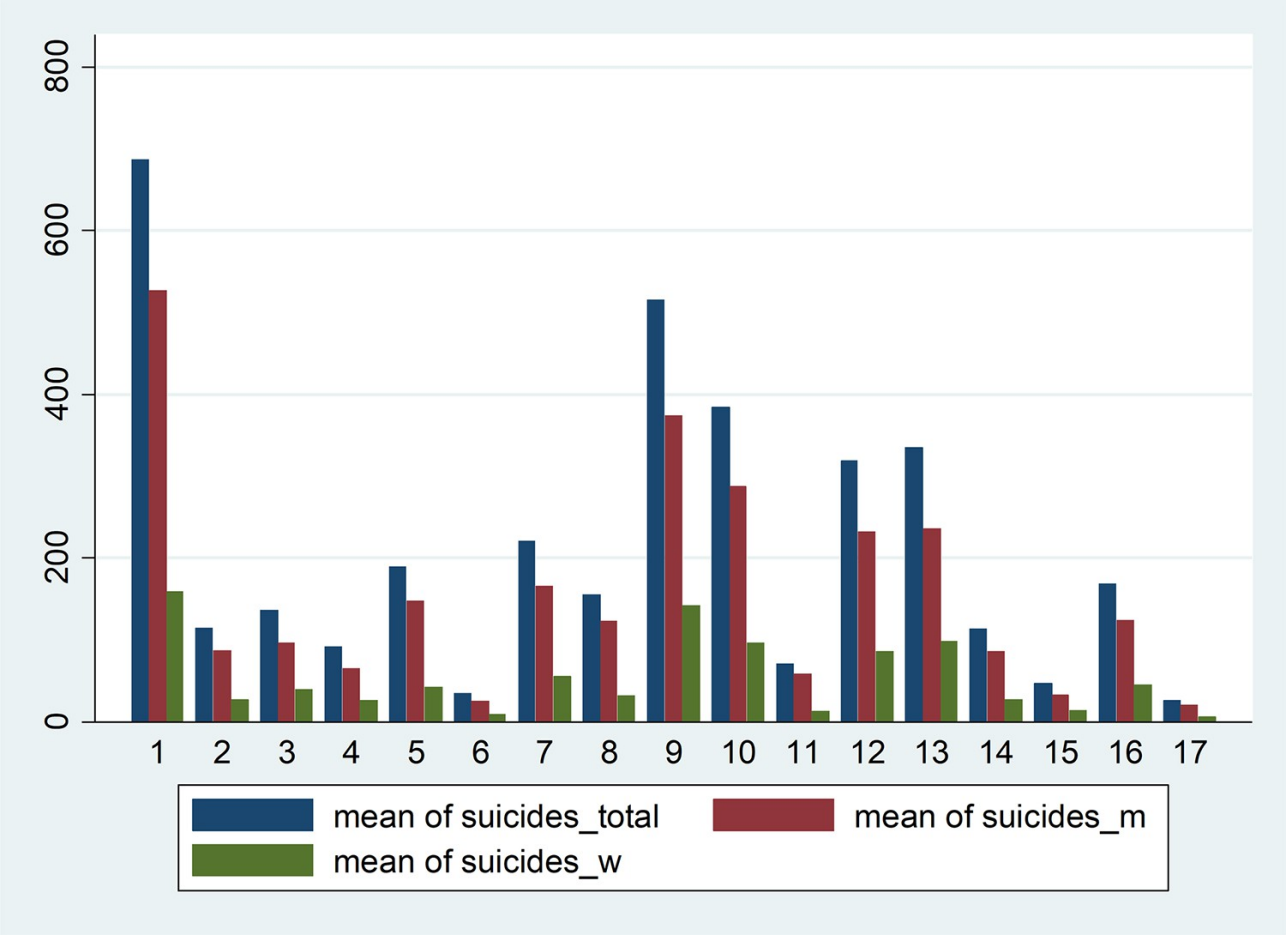
Distribution of suicides (mean 2014–2019) by region. *Notes*: Andalusia (region = 1), Aragon (region = 2), Asturias (region = 3), Balearic Islands (region = 4), Canary Islands (region = 5), Cantabria (region = 6), Castile and Leon (region = 7), Castile-La Mancha (region = 8), Catalonia (region = 9), Valencian Community (region = 10), Extremadura (region = 11), Galicia (region = 12), Madrid (region = 13), Murcia (region = 14), Navarre Community (region = 15), Basque Country (region = 16), and La Rioja (region = 17). *Source*: Authors’ elaboration.

Besides, Fig 1 plots the time evolution of total suicides by region, whereas Fig 2 shows the distribution disaggregating by sex.

From this first approximation to our data, a slightly pattern is observed: higher suicide rates are for males and Mediterranean areas.

Specifically, Table 3 presents the empirical results (n = 102). In the first column, results for the total number of suicides are presented whereas the latest two contains the ones for males and females, respectively.

**Table 3:** Poisson/Negative binomial panel results.

Variable	*suicides_total*		*suicides_m*		*suicides_w*	
*foral*	-0.607		-0.495		-0.832	
	(-1.09)		(-0.87)		(-1.34)	
*north*	0.363		0.275		0.472	
	(0.80)		(0.61)		(1.04)	
*mediterranean*	0.489		0.646		0.769	*
	(1.17)		(1.58)		(1.82)	
*unemployment*	0.000		0.007	**	0.001	
	(-0.12)		(2.24)		(0.21)	
*at-risk-of-poverty*	-0.007		-0.007		-0.012	
	(-1.55)		(-1.42)		(-1.49)	
*density*	0.002	*	0.002	**	0.001	*
	(1.83)		(2.16)		(1.74)	
*isolation*	-0.072	**	0.029		-0.065	*
	(-2.10)		(1.03)		(-1.71)	
Alpha *p*-value	0.000		0.000		0.000	

Source: Authors’ calculations.

*Notes*: *z*-statistics in parentheses. ***, **, and * denote significant at 1%, 5%, and 10% respectively. Observations: 102. The use of Poisson or the negative binomial estimator is determined by the Alpha parameter. If Alpha *p*-value < 0.05 it is estimated the negative binomial model.

It is noteworthy from Table 3 that coefficients are somewhat statistically significant and in most cases have the expected signs according to the priori economic criteria: (i) self-regional area characteristics and material deprivation factors, when significant (Mediterranean and unemployment, but not North, “foral” or the percentage of population that is at risk of poverty), have a clear positive effect on suicides, (ii) the urban-rural pattern is obtained (through density, being about 2 points higher), (ii) social isolation, has the reverse expected sign (our empirical results indicate that it would reduce the number of suicides by around 7 percent).

However, it can be observed that there appear differences regarding gender. That is, “bioclimatic theory” is obtained for women (about 77 percent more times) whereas unemployment, during economic expansions, tend to raise its importance and are higher for men than for women (by 0.7 percent). Another important fact is the resilience for women living alone as a natural outcome (around 7 percent lees times).

## Discussion and conclusions

The incidence of suicide in developed countries is an important public health issue and its nonstop rise is puzzling scholars and policymakers [18]. Thus, in recent years, there have been several studies on the risk factors for suicide and self-harm. An emerging claim has thus arisen concerning the need to reduce them.

As well, what has been proved regarding macroeconomic conditions and suicides is that better economic conditions are traditionally associated with lower suicides. That is, they appear to be counter-cyclical [14]. However, over recent years, there is also empirical evidence suggesting that suicide rates may not necessarily behave in a counter-cyclical way. Besides, since the Great Recession of 2008, there has appear new interest by studying economic uncertainty [19–21].

We believe that our findings are consistent with those studies that address the potential impact of economic conditions [8,20] and/or social isolation on suicides [22] while considering gender differences [23]. Our empirical results confirm that the profile of a person who commits suicide would be the following: an unemployed man living in an urban area or a woman who live not alone in an urban Mediterranean area.

Indeed, regarding our initial hypotheses, we first show that there is a substantial, significant link between suicides and regional, material deprivation and urbanization/rural factors in Spain. Secondly, that social isolation factors characteristics matter in suicides. Thirdly, that there are significant gender differences.

Our findings have powerful public health policy implications. Our study supports the notion that public intervention is needed to reduce suicide and self-harm [24]. Specifically, urban-rural disparities in suicides. Furthermore, taking into account the diversity between women and men, to consider different strategies stratified by gender. All in all, education programs and/or improvements in housing conditions could be also beneficial. In addition, social support may have an important protective effect against suicidal behavior.

However, our study has certain limitations. Mainly, as discussed previously, we work with aggregated data. Hence, recommendations and policy implications should be taken with caution. Finally, as an extension of this study it would be interesting to explore the results in pandemic COVID-19 period as data become available being crucial to explore more possible mechanisms.

## Supporting information

S1 File Anonymized data set. (DTA)
